# High prevalence of overweight and obesity among a representative sample of Puerto Rican children

**DOI:** 10.1186/s12889-015-1549-0

**Published:** 2015-03-05

**Authors:** Augusto R Elías-Boneta, Milagros J Toro, Omar Garcia, Roxana Torres, Cristina Palacios

**Affiliations:** School of Dental Medicine, Medical Sciences Campus, University of Puerto Rico, Rio Piedras, Puerto Rico; School of Public Health, Medical Sciences Campus, University of Puerto Rico, Rio Piedras, Puerto Rico

**Keywords:** Overweight, Obesity, Prevalence, Children, Puerto Rico

## Abstract

**Background:**

The prevalence of childhood overweight and obesity has become a public health problem worldwide. The objectives of the study were: 1) to establish the BMI prevalence in 12-year olds residing in Puerto Rico, and 2) to determine BMI differences by sex, public-private school type, and geographic regions.

**Methods:**

Data was obtained from an island-wide probabilistic stratified sample of 1,582 twelve-year-olds (53% girls and 47% boys). The BMI was determined using the National Health and Nutrition Examination Survey procedures. Children were categorized as underweight, healthy weight, overweight or obese using the Center for Disease Control and Prevention’s age and gender specific growth charts. A logistic regression model was used to estimate BMI category prevalence. Odds ratios were calculated using a multinomial regression.

**Results:**

In this study, 18.8% of the children were overweight and 24.3% were obese. A higher prevalence of obesity was observed in boys as compared to girls, 28.2% vs. 20.2%, respectively. The estimated prevalence of overweight and obesity in children from public schools was lower than for those from private schools. After adjusting for type of school and region, boys had a significantly higher risk of being obese (64%) as compared to girls. In public schools, boys had a lower prevalence of being overweight while girls had a higher prevalence compared to children attending private schools. Girls attending private schools had a higher obesity prevalence (27.8%) compared to girls from public schools (19.8%). The prevalence of underweight (2.7%) is slightly lower than in the United States.

**Conclusions:**

The prevalence of overweight and obesity of 12-year-olds residing in PR was 18.8% and 24.3%, respectively; higher than in the U.S. (by groups). Boys were at higher risk of obesity than girls. There is an urgent need to implement public health policies/programs to reduce the prevalence of overweight and obesity in children in PR.

## Background

Childhood obesity is a serious public health problem worldwide [1-3] affecting low and middle income families [4]. During childhood and adolescence, being obese or overweight may lead to physical, psychological, and social health problems [5-11]. These health conditions may persist through adulthood [12], contributing to a significantly lower health-related quality of life [13]. Moreover, obesity/overweight associated-health conditions are related to the primary causes of mortality worldwide [14-16]. The burden of cardiovascular disease (CVD) for Puerto Ricans is substantial. Cardiometabolic risk refers to the probability of having diabetes, heart disease or stroke [17]. For U.S. residing Puerto Ricans approximately 18% of the adults had diabetes, 28% hypertension, and 45% hypercholesterolemia, as examples of metabolic conditions [18]. For Puerto Ricans residing in the island, 13.5% self-reported diabetes, 37% had hypertension, and 38% hypercholesterolemia, among other CVD factors [19].

The worldwide prevalence of childhood overweight and obesity increased 10% between 1990 and 2010 [20]. During the last 30 years, childhood obesity in the US has more than doubled and has quadrupled in adolescents [21,22]. The 2011–2012 National Health and Nutrition Examination Survey (NHANES) [22] states that 34.5% of the 12–19 year-olds were obese or overweight and 20.5% were obese. In this report, obesity was higher among non-Hispanic black and Hispanic children as well as adolescents compared to non-Hispanic white children. Obesity rates of Puerto Rican children in mainland US and in PR are among the highest (24-36%) when compared to other racial/ethnic groups [23]. An island-wide study conducted among 3,079 second graders established the prevalence of at risk of overweight at 16% and of overweight at 26% [24]; these categories are currently termed overweight and obese, respectively [25]. In another investigation, the combined self-reported prevalence of overweight and obesity in 10–19 year-olds in PR was 34.9% while in 10–14 year-olds was 52.3% [26]. Moreover, a study of 250 elementary school children in PR revealed that 26.8% were obese and 11.3% were overweight [27]. These previous studies employed convenient samples of limited size in specific communities except the study conducted among second graders [24], which was published as a government report in Spanish.

In the U.S., Healthy People 2020 established targets to guide the health promotion and disease prevention efforts [28] and to reduce obesity in children and adolescents [29]. In PR, the only island-wide study, on childhood obesity was conducted in 2005 [24]; therefore, no ongoing surveillance program exist posing a challenge to monitor changes and control these conditions. The objectives of the study are: 1) to establish the BMI prevalence in 12-year olds residing in Puerto Rico, and 2) to determine BMI differences by sex, public-private school type, and geographic regions.

## Methods

### Study and sample design

The present study was an ancillary, island-wide cross-sectional study designed to examine oral health status of 12-year-olds residing in PR. Although boys and girls aged 12 are at a different pubertal stage, the World Health Organization recommends this age group to establish international oral health comparisons [30], which was the primary goal of this investigation. The sample design allows the inference for the population of all 12 year-olds enrolled in school during the study period (November 2010 to May 2011). According to the PR General Council on Education and the Department of Education, there were 51,376 (40,081 in public and 11,295 in private schools) 12-year-olds enrolled in school. Public and private schools, serving fifth to seven graders in PR during the study period, were used as the target sample.

### Sample selection

A probabilistic sample of 133 schools was selected from a total of 1,843 schools (1,343 public and 500 private schools). The sample is proportional to enrollment size, and stratified by the 11 health administrative regions (as determined by “Reforma”, the PR government’s health insurance program in 1997) [31], type of school (public and private), and gender. Ten boys and ten girls were randomly selected from each school using the official student lists provided by the school’s administration. The required sample size was 2,600 children. A total of 1,734 children were contacted and invited to participate, however, some of the selected schools were inactive and others had merged. One hundred-fifty two (152) children were excluded due to school absences and failure to obtain their parent’s written consent.

### Inclusion/exclusion criteria

The inclusion criteria: 12-years-of-age at the time of recruitment; healthy (no history of medical problems that contraindicate their participation in the study). The exclusion criteria: not competent to give their consent or unable to withstand a clinical procedure.

Parents or guardians signed a written informed consent and children provided written assent approved by the Institutional Review Board of the Medical Sciences Campus of the University of PR.

### Socio-demographics and anthropometric measurements

Socio-demographic data was obtained from a self-reported medical history questionnaire answered by the legal guardian. In PR, public/private school attendance may be considered a surrogate measure for the family’s socioeconomic status [31], which was confirmed in a subset sample [32]. In addition, it has been reported that over 86% of the students attending public school in PR are socio-economically disadvantaged [33], thus the type of school attended was interpreted as a proxy of the participant’s socio-economic status. Trained staff measured children’s height and weight following the NHANES III procedures [34]. Height was measured in meters using a portable stadiometer (Seca Corporation, Hanover, MD) and weight was measured in kilograms using a Body Composition Analyzer (TBF-310A, Tanita Corporation of America, Arlington Heighs, IL), calibrated prior to each use. These anthropometric measurements were made while the children were wearing their uniforms (without socks, shoes, and accessories). Body Mass Index (BMI) was calculated using the following formula: weight (kg)/height (m)^2^.

Children were categorized as underweight, healthy weight, overweight, or obese using the Center for Disease Control and Prevention's age and gender specific growth charts [35], as follows: underweight (BMI <5^th^ percentile), healthy weight (BMI between the 5^th^ percentile and <85^th^ percentile), overweight (BMI between >85^th^ percentile and <95^th^ percentile), and obese (BMI > 95^th^ percentile) for children of the same age and gender [36].

### Statistical analysis

The overall estimated prevalence and prevalence odd ratios (*POR*) for the different BMI categories were calculated. The overall estimated prevalences were also calculated by socio-demographic variables (gender and type of school) and their interactions for each weight category were estimated using a logistic regression model [37] as follows:

$$ {P}_i=\frac{1}{1+{e}^{\left( Bo+{B}_i{X}_i\pm 1.9SE\right)}} $$

describes the estimator of the weight category;

*β*_0_ is the model intercept, x_i_ is a socio-demographical variable;

and *β*_*1*_ is a change for each category.

In addition, a descriptive analysis by region was conducted using the 11 health administrative regions.

*POR* were calculated using a multinomial logistic regression to evaluate association between the weight category (overweight or obese) and socio-demographic variables (gender, type of school, and school zone). The base outcome for these models was normal weight. The 11 health regions were collapsed into 3 zones for the *POR* calculation based on the diet intake differences among these zones [32]: Coastal, Metropolitan, and Central Mountain. A robust variance estimator was used to determine the standard errors of the prevalence parameters due to the school effect. All statistical analyses used robust variance estimators to determine the standard errors of the prevalence parameters for intraclass correlation within school. Weights were assigned to each student by using the inverse of the probability of selection of the sampling design. All statistical analyses were conducted by incorporating the sampling weight to obtain unbiased estimates from the sampling design, using the statistical package Stata (Version 12.0, College Station, TX, USA). A probability value of 0.05 was considered statistically significant.

## Results

A total of one thousand five hundred and eighty two (n = 1,582) subjects were evaluated between November 2010 and May 2011. The distribution of the demographic characteristics of gender and type of school attended were as follows: 53% females, 47% males; 77.2% public schools, 22.8% private schools. The distribution in relation to the school’s region was: North (10.5%), Northwest (4.9%), Northeast (6.0%), East (8.2%), Southeast (9.0%), Southwest (5.2%), Ponce (4.3%), West (4.9%), Metro area (22.8%), San Juan (10.3%) and Mountain (14.0%). By zones, the distribution was: Coast (n = 837; 52.9%), Metropolitan (n = 523; 33.1%) and Central Mountain (n = 222; 14.0%).

The estimated prevalence and 95% confidence intervals (CI) for each BMI category are shown in Table 1. Overall, the prevalence of obesity was 24.3% (CI: 19.9%, 29.2%) and for overweight it was 18.8% (CI: 16.4%, 20.3%). A higher prevalence of obesity was seen in boys (28.2%, CI: 18.7%, 40.1%) than in girls (20.2%, CI: 19.5%, 29.2%). In terms of school type, children in public schools had a lower prevalence of overweight (18.6%, CI: 15.4%, 22.4%) and obesity (24.0%, CI: 19.5%, 29.2%), when compared to children in private schools (overweight 21.2%, CI: 16.4%, 27.1%; obese 28.2%, CI: 18.7%, 40.1%).

**Table 1:** **Estimated Prevalence and 95**% **CI of Underweight, Healthy Weight, Overweight, and Obesity by Gender and School Type**

		**Underweight**		**Normal weight**		**Overweight**		**Obese**	
		**Weighted** ^**1**^	**Unweighted**	**Weighted** ^**1**^	**Unweighted**	**Weighted** ^**1**^	**Unweighted**	**Weighted** ^**1**^	**Unweighted**
Overall **n=1,582**		2.7% (1.8, 4.1)^2^	3.0% (2.3, 4.0)	54.3% (50.2, 58.3)	55.5% (53.0, 57.9)	18.8% (16.4, 20.3)	18.3% (16.4, 20.3)	24.3% (19.9, 29.2)	23.2% (21.2, 25.3)
		**n=48**		**n=878**		**n=289**		**n=367**	
Gender	Boys **n=744**	2.7% (1.7, 4.4)	3.9% (2.7, 5.6)	50.1% (44.2, 56.0)	52.7% (49.1, 53.7)	18.5% (14.9, 22.6)	17.3% (14.8, 20.2)	28.2% (18.7, 40.1)	26.1% (23.1, 29.4)
		**n=29**		**n=392**		**n=129**		**n=194**	
	Girls **n=838**	2.7% (1.6, 4.4)	2.3% (1.5, 3.5)	58.1%(53.1, 62.9)	58.0% (54.6, 61.3)	19.0% (14.6, 22.3)	19.1% (16.4, 21.8)	20.2% (19.5, 29.2)	20.6% (18.0, 23.5)
		**n=19**		**n=486**		**n=160**		**n=173**	
School Type	Public **n=1,221**	2.7% (1.8, 4.2)	3.3% (2.4, 4.4)	54.6% (50.3, 58.8)	56.4% (53.5, 59.1)	18.6% (15.4, 22.4)	18.6% (15.4, 22.4)	24.0% (19.5, 29.2)	22.4% (20.1, 24.8)
		**n=40**		**n=688**		**n=220**		**n=273**	
	Private **n=361**	2.1% (1.0, 4.3)	2.2% (1.1, 4.4)	48.5% (35.9, 61.3)	52.6% (47.5, 57.7)	21.2% (16.4, 27.1)	19.1% (15.4, 23.5)	28.2% (18.7, 40.1)	26.0% (21.8, 30.8)
		**n=8**		**n=190**		**n=69**		**n=94**	

^1^weighted by the inverse probability (1/*p*) of the selection.

^2^95% Confidence Interval (CI).

Table 2 shows the estimated prevalence of overweight and obesity stratified by type of school and gender. In private schools, more boys were overweight (26.6%, CI: 15.3%, 38.1%) than in public schools (17.9%, CI: 13.9%. 21.9%). Girls from private schools had a lower prevalence of overweight (15.1%, CI: 8.97%, 21.3%) than those in public schools (19.2%, CI: 14. 8%, 23.7%); while obesity prevalence in girls from private schools (27.8%, CI: 15.9%, 39.7%) was higher compared to those attending public schools (19.8%, CI: 15.4%, 24.3%). This difference was not observed in boys.

**Table 2:** **Estimated Prevalence of Underweight, Healthy Weight, Overweight, and Obese by Gender and School Type**

**BMI Categories**		**School type**			
		**Public (n = 1,221)**		**Private (n = 361)**	
		**Weighted** ^**1**^	**Unweighted**	**Weighted** ^**1**^	**Unweighted**
Underweight **n = 48**	Boys **n = 29**	2.8% (1.37, 4.20)^2^	4.4% (2.89, 5.96)	2.0% (0.035, 4.01)	2.2% (0.32, 4.15)
		**n = 25**		**n = 4**	
	Girls **n = 19**	2.7% (1.27, 4.11)	2.3% (1.23, 3.33)	2.2% (−0.17, 4.55)	2.2% (1.72, 4.22)
		**n = 15**		**n = 4**	
Healthy Weight **n = 878**	Boys **n = 392**	50.6% (44.38, 56.87)	53.6% (49.20, 58.05)	42.8% (24.84, 60.72)	49.7% (41.17, 58.27)
		**n = 303**		**n = 89**	
	Girls **n = 486**	58.2% (53.04, 63.44)	58.7% (54.62, 62.76)	54.9% (42.17, 67.66)	55.5% (48.47, 62.52)
		**n = 385**		**n = 101**	
Overweight **n = 289**	Boys **n = 129**	17.9% (13.94, 21.92)	16.4% (13.5, 19.34)	26.7% (15.25, 38.07)	20.1% (13.93, 26.29)
		**n = 93**		**n = 36**	
	Girls **n = 160**	19.2% (14.75, 23.74)	19.4% (16.45, 22.29)	15.1% (8.97, 21.27)	18.1% (13.34, 22.92)
		**n = 127**		**n = 33**	
Obesity **n = 367**	Boys **n = 194**	28.7% (21.76, 35.57)	25.5% (21,79 29.18)	28.5% (16.63, 40.44)	27.9% (20.11, 35.75)
		**n = 144**		**n = 50**	
	Girls **n = 173**	19.8% (15.35, 24.30)	19.7% (16.13, 23.01)	27.8% (15.87, 39.68)	24.2% (17.96, 30.39)
		**n = 129**		**n = 44**	

^1^weighted by the inverse probability (1/*p*) of the selection.

^2^95% Confidence Interval.

Boys were at a significantly higher risk (POR _weighted_ =1.64, CI: 1.18, 2.35) of being obese compared to girls, after adjusting for type of school and region. No other statistical differences were observed (Table 3).

**Table 3:** **Weighted Prevalence Odd-ratios** ***(POR***_***weighted***_ ***)*** **for obese or overweight vs. healthy weight and socio-demographic variables (n = 1534)** ^**1**^

		**Overweight** ^**2**^		**Obese** ^**2**^	
		**Crude**	**Adjusted** ^**3**^	**Crude**	**Adjusted** ^**3**^
**Gender**	Boys^4^	1.12 (0.80, 1.58)	1.11 (0.79, 1.57)	1.64 (1.18, 2.29)	1.66 (1.17, 2.35)
**School type**	Public^5^	0.78 (0.47, 1.30)	0.81 (0.50, 1.32)	0.76 (0.38, 1.50)	0 .72 (0.37, 1.42)
**Zone**	Metro^6^	1.23 (0.604, 2.51)	1.20 (0.58, 2.46)	0.74 (0.33, 1.65)	0.65 (0.31, 1.37)
	Coast^6^	1.20 (0.56, 2.57)	1.18 (0.55, 2.56)	0.67 (0.32, 1.39)	0.69 (0.30, 1.58)

^1^Underweight Children were excluded of the multinomial logistic regression models.

^2^Reference outcome (normal).

^3^Adjusted by gender, type of school, and region.

^4^Reference category: girls.

^5^Reference category: private school.

^6^Reference category: Center/Mountain Zone.

Children in the East, Northeast and Southwest regions had the highest estimated prevalence of overweight (≥19.5%), while the Southeast and Northwest regions had the lowest (14.1% and 14.3%, respectively). The highest obesity prevalence was found in the Northeast region (33.7%), followed by Northwest region (25.9%), and Ponce (25.0%). The lowest was observed in the Mountain and Southwest regions (19.4% and 19.5%, respectively).

## Discussion

The aim of this study were to establish the BMI prevalence in 12-year olds residing in Puerto Rico, and to determine BMI differences by sex, public-private school type, and geographic regions.. We found that the overweight prevalence was 18.8%, which is higher than those reported in prior studies in Puerto Rican children aged 7 years (16.2%) [24] and 9–10 years (11.3%) [27]. The overall prevalence of obesity was 24.3%, which is slightly lower than the prevalence reported in the aforementioned studies in PR (25.7% and 26.8%) [24] [27]. The obesity prevalence in PR, as presented by this study, is higher than the overall prevalence in U. S. for 12-19-year-olds (20.5%) and higher when stratified by race/ethnicity (whites 19.6%, non-Hispanic blacks 22.1%, and Hispanics 22.6%) [38]. Overweight prevalence in the U.S. was presented together with obesity data, precluding its comparison [38]. The prevalence of underweight in this study (2.7%) is slightly lower than in the US (3.6%) [39].

The current study portrayed boys with a higher prevalence of obesity but similar prevalence of overweight when compared to girls while boys were at significantly higher risk of being obese compared to girls. A previous study in PR found that boys had a higher prevalence of obesity compared to girls (26.9% vs. 24.5%); however, the overweight prevalence for girls was higher than for boys (17.4% vs. 14.8%) [24]. This finding is similar to a recent study in 436 Puerto Ricans aged 10–19 years [26], whereas it was reported that females were 50% less likely than males to be overweight or obese. The current study’s results are consistent with US national data from NHANES 2009–10, where male children and adolescents from all racial/ethnic groups had higher obesity prevalence compared to females [40].

The obesity prevalence data from NHANES 2011–12 [22] showed no differences in gender when all races/Hispanic origin groups were considered. The exception was between Hispanics and non-Hispanic Asian boys, who had higher obesity prevalence when compared to girls; however, these differences were not statistically significant. Worldwide data indicates that adolescent boys have higher prevalence of obesity in almost all nations and higher prevalence of overweight in almost half of the countries compared to girls [3]. Weight differences between boys and girls may be related to awareness of weight control and to body image issues that may develop in pre-adolescence [41].

Regarding the type of school attended, children from private schools had a higher prevalence of overweight and obesity than those attending public schools. The results are consistent with those of the 2005 island-wide study [24] where children attending private schools had a higher BMI than those attending public school. It is possible that children who attend private school, a surrogate (indication?) to higher socioeconomic status in PR, have higher purchasing power to consume sweets, snacks, and fast foods more often. These findings may be also attributed to the existence of the National School Lunch Program (NSLP) in the public school system, which is not available in most private schools. It has been reported that children participating in the NSLP have lower prevalence of overweight and obesity than non-participating children [42]. NSLP provides nutritionally balanced meals for lunch, which could be beneficial in reducing the risk of overweight and obesity.

Overall, we observed a higher risk of obesity in boys as compared to girls. When stratified by gender and type of school, the study suggests a high prevalence of obesity in boys in both public and private schools; however, girls from private schools had higher obesity prevalence than those in public schools. In contrast, the percentage of overweight girls attending public school was higher but no significantly different than those attending private school. Similarly, the study conducted in Cayey, PR, reported a higher prevalence of obese/overweight in girls from private schools compared to girls from public schools [27]. Another study in children and adolescents attending public schools in the San Juan Metropolitan Area reported that girls had a better diet compared to boys due to their higher participation rate in the NSLP [43]. Therefore, participation in the NSLP program provides an alternative explanation for the weight differences between boys and girls in PR.

A recent study conducted in the San Juan Metropolitan area found that girls had significantly higher scores for whole fruits and vegetables than boys [44]. In addition, overweight/obese children had a significantly lower availability of unhealthy foods at home, higher access to home recreational/sport facilities, lower use of school recreational/sport facilities, but reduced participation in school breakfast programs (SBP). The authors concluded that all participants had either poor diet or a diet that was not adequate [45]. Although the diet data was obtained by interviews with trained nutritionist, the availability and access to healthy or unhealthy foods and to sports and recreation was obtained through self-reported nutrition questionnaires. More research is needed to assess the factors associated with overweight/obesity and to assess the contribution of the SBP, NSLP, and other dietary habits on caloric intake and meal quality offered in private schools and health regions.

Our study revealed that the highest obesity prevalence was found in the Northeast, Northwest, and Ponce regions of PR, and the lowest prevalence in the Mountain and Southwest regions. Our findings are in partial agreement with an island-wide study conducted in a representative sample of second graders. In this study, the highest obesity prevalence was found in the municipalities located in the Eastern and Northern regions; the lowest prevalence was observed in the Metropolitan area [24]. Body weight differences among regions may be due to local environmental factors such as the availability of healthy and unhealthy foods and food outlets as well as the accessibility of recreational/sports facilities and physical activity programs at home and school [46-48]. Unfortunately, the difference in the number of health regions and their geographical boundaries prevent an in-depth comparison between the two studies.

According to the 2010–11 U.S. Census, 45.6% of Puerto Ricans households fall below the poverty level [49]. Health inequalities exist and persist between Puerto Ricans residing in the island and US citizens in the mainland. For example, even though the prevalence of obesity/obesity-related diseases is higher in PR [50], the island does not have an ongoing surveillance program to project 2020 obesity trends in school-age children. Considering NHANES historical trends in obesity (1971–2008), US children are projected in 2020 to be 1.8 kg heavier than a child in 2007–2008, and adolescents to be 2.7 kg heavier than adolescents during this period. It has been estimated that a child should reduce his/her caloric intake by 164–286 kcal/day, depending on their race-ethnicity, to achieve the goals of Healthy People 2020 [51]. Currently, the daily caloric intake required to meet these goals cannot be determined in PR. To define the appropriate reduction in caloric intake for Puerto Rican children, it is necessary to determine obesity trends and future projections. Risk factors including diet, physical activity, and other behavioral, social and physical environmental health determinants need to be investigated to better understand the complexity of obesity in Puerto Rican children to reduce this gap. Further investigation is needed to determine the social, physical and environmental factors related to this public health problem, allowing designing suitable policies and programs to reduce the prevalence of overweight and obesity.

One of the strengths of this study is the inclusion of a large and representative sample of 12-year-olds residing in PR. The interviewers and personnel conducting the anthropometry were trained using standardized methods. One of the limitations in regards to obesity is that the sampling was designed to estimate oral health outcomes; in consequence, important risk factors including dietary/snacking habits and physical activity of the child, as well as potential parental correlates, e.g., weight/obesity status were not investigated as study variables. However, with no contemporary anthropometric data on children in PR, island-wide, population-base BMI data is of importance for the public health community. Further investigation of these important obesity risk factors is required. When stratified by gender and type of school/region, some of the strata had limited number of subjects. Since BMI is not a good indicator of visceral obesity, future studies should include other anthropometric measurements including waist circumference, a simple and inexpensive method.

This island-wide study provides current data on the magnitude of the childhood overweight and obesity epidemic in PR. Our findings may contribute in mobilizing resources for the design of studies to identify successful policies and interventions.

## Conclusions

The prevalence of overweight and obesity of 12-year-olds in PR was 18.8% and 24.3%, respectively. These values are higher than those reported by NHANES in US children. Boys are at a higher risk of overweight/obesity when compared to girls.
